# Editorial Chit Chat

**Published:** 1871-07

**Authors:** 


### EDITORIAL CHIT CHAT.

OUR NEW QUARTERS.—We hope to be able to occupy our new apartments, at the old place, by the first of August. When completed, they will be the best fitted and best arranged Surgical Rooms in the country.

DARWINISM.—Darwin's views are evidently gaining a foothold in our city. We are informed that a lady made application, to one of our dressmakers, for a pattern for a "Gorilla" basque. She was supplied with a "gabrielle," which answered as well.

A VERY SNUG MEDICAL FEE.—When the Prince of Russia lay dying at Nice, Prof. Oppolzer, of Vienna, was summoned by telegraph, but arrived too late—the Prince was dead. But the Emperor met the Prof. with his carriage at the station, and besides paying him the honor due a Prince, paid him also the sum of twenty-five thousand guldens, or $13,887.50.

UTILITY OF CANADIAN CENTS.—It is perhaps not generally known that the Canadian cent is a reliable standard of weight, measure and value; one hundred of them weighing a pound, each one measuring an inch in diameter, and one hundred making a dollar. Of course there are ten to a dime, twelve to a foot, and twenty-five to a quarter of a pound.

RULLOFF.—We have read several amusing accounts of the examination of Rulloff, relative to his sanity, by Drs. Gray and Vanderpoel. Several exchanges assure us that Rulloff did not believe in the existence of a God, that he did not accept the truths of Christianity, and that "*therefore*, they are of the opinion that Edward H. Rulloff is in sound physical health and entirely sane." This is the first instance we have ever known of a verdict of sanity being given *because* a man did not believe in God and Christianity.

"RULING PASSION, &c."—A friend who recently lost heavily, by means of a fire which destroyed a manufactory, in which he was largely interested, and which was located on the banks of a trout stream, proposed to us, after having summed up his loss, that we go fishing, and roast our trout in the hot ashes of the ruins. That's what we call a philosophical view of a disaster.

---

THE GERMAN LANGUAGE.—Dr. White, Prof. of Chemistry in Harvard University, in an "Introductory Lecture before the Medical Class," advises students to study the German language on account of its importance. Nine-tenths of all that is new and important in physiology, pathology, and medical chemistry, is the work of German hands and brains. If the student wishes to keep up with the progress of his art, he must learn German.

---

EXCURSION OF ST. OMER'S.—St. Omer's Commandery of Knights Templar, of this city, intend visiting the Grand Commandery of the State of Maryland, at their annual conclave, in the city of Baltimore, in September. The Maryland Sir Knights will have the pleasure of witnessing the evolutions of the best drilled Commandery in this State. Commander WILLIAMS never allows himself to be outdone by anybody in Masonic matters, and the Sir Knights of his command always second his motion.

---

ELEGANT PLACE.—A. Wyckoff, Esq., who has done so much toward beautifying our city, in the erection of beautiful buildings, is now engaged in building, in the Fifth Ward, the finest residence and grounds in the city. He has purchased all the land between his old residence and the Rail Road, and is carting Clinton Island upon it, over a pontoon bridge, built for the purpose. He intends not only to be high and dry, but to have elegant grounds as well.

---

"SOUTHERN TIER ORPHANS' HOME."—The second number of this neat little quarterly has been received. Mrs. Luther Caldwell, the able and accomplished Editress, is making an exceedingly interesting journal of it. It is devoted to the interests of the Orphans' Home; fifty cents a year is asked for it, and should find a subscriber in every family in the Southern Tier. Send in your subscription to Mrs. Luther Caldwell, Elmira, N. Y., and help a good cause.

A NEW BITTERS.—Our friend Sanders is the inventor of a new "vinegar bitters," which promises to supercede all other quack medicines. He *sold* Dr. Gregg a sufficent quantity recently, to constitute him wholesale agent for the same. Jeff. Wisner, the N. C. R. W. man promises to become traveling agent for the new beverage.—For further particulars enquire of the Doctor at his drug store.

---

SURGICAL INSTRUMENTS.—Geo. Tiemann & Co., of New York, have just manufactured for us the finest general operating case of instruments, owned by one person, in the U. S. It is complete in all its appointments, containing everything of value to the surgeon. The instruments are beautifully finished, of the latest and most approved designs, and of the best materials. We are sure there is not a more complete set in this country, and being made by Geo. Tiemann & Co., is sufficient guarantee that there are none better.

---

THE TITLE OF "DOCTOR."—The title of "doctor" was invented in the twelfth century. Irnerius, a learned professor of law at the University of Bologna, induced the Emperor Lothaire II, whose chancellor he was, to create the title, and he himself was the first recipient of it. He was made doctor of laws of that University. Subsequently the title was borrowed by the faculty of theology, and first conferred by the University of Paris on Peter Lombard. William Gordenio was the first person upon whom the title of doctor of medicine was bestowed; he received it from the College of Asti, in 1329.

---

POPULAR ERRORS.—To think that the more a man eats the fatter and stronger he will become. To believe that the more hours children study the faster they will learn. To conclude that, if exercise is good, the more violent it is the more is done. To imagine that every hour taken from sleep is an hour gained. To act on the presumption that the smallest room in the house is large enough to sleep in. To argue that whatever remedy causes one to feel immediately better is good for the system, without regard to more ulterior effects. To eat without an appetite, or continue to eat after it has been satisfied, merely to gratify taste. To eat a hearty supper for the pleasure experienced during the brief time it is passing down your throat, at the expense of a whole night of disturbed sleep, and a night of weary waking in the morning.

ARTIFICIAL LIGHT.—Much injury is done the eye by the improper use of lamp or gas light. Many persons suppose that a bright light, in reading, is injurious to the eye, and therefore attempt to read with a low, unsteady light. This is wrong. Let the light be bright and steady—the brighter the better, but let it be placed so that it may not shine directly into the face, but over the shoulder. No harm is done by illuminating the book well—the error is in allowing the light to fall directly in the face. Gas light is better than lamp light, if it is steady and brilliant. The student's Lamp is the best kerosene light for reading purposes. Remember to sit with your back to the light, then let it burn brightly and illuminate your book well.

---

AIR YOUR BEDS.—Housekeepers are not sufficiently careful in airing beds and sleeping apartments. During sleep there is constantly going on a sort of insensible perspiration of the body, through which the impurities of the system are thrown off, and are absorbed by the bed-clothing. In these beautiful mornings the clothing should be removed from the bed, carefully spread over chairs, and placed next the open windows for free ventilation. Occasionally the mattress should be thrown upon the roof, or upon the grass where the sun may shine upon it for a few hours. In this manner bed and bedding can be kept pure and cleanly. It is very unhealthy to sleep in a bed repeatedly, without this system of ventilation.

---

A FREE PUFF.—Recently we had occasion to spend a few days in New York City. In order to be near some friends, we were recommended to stop at the "Ashland House," on 24th street and 4th Avenue. The house is kept by gentlemen by name of Brockway, who are very unfortunate in their selection of servants. They have placed their Restaurant in charge of a big, double-fisted, short-haired, bull-headed Irishman, who would be more in place in a fourth rate whisky saloon, instead of being entrusted with the orders of gentlemen who may visit the establishment for their meals. The Cashier is a red-whiskered, bald-headed puppy, who takes more delight in insulting the guests of the house than in receiving their money. We advise persons visiting New York to steer clear of the Ashland House, until the Messrs. Brockway replace their steward and cashier by gentlemen, instead of bullies.

THE WAY TO BUILD A CHURCH.—The Congregational Society of this city have been talking up the matter of a new meeting house. Their Pastor—the Rev. T. K. Beecher—thinks the best way to build a church is to have its members contribute the necessary funds, without urging or begging in any form. Accordingly he prepared several hundred blanks, and left them where all, who felt so disposed, could get them and write thereon the amount they would cheerfully give toward the new edifice,—such subscription to be known to no human person, save the minister. About one half the blanks have been returned, and Mr. Beecher announced, last Sabbath, that the amount subscribed already reached $50,253. Truly, a beautiful and sensible way for any christian people to build a meeting house.

---

PROMPT SETTLEMENT.—In the recent burning of our Eye and Ear Institute, we were fortunate in being insured in two reliable Companies, by which means three thousand dollars of our loss was repaid to us. We held a policy of fifteen hundred dollars in the MARKET INSURANCE Co. of New York, and a like sum in the NATIONAL of Boston, represented in this city by Ayres & Barney. Three days after our proofs of loss were submitted to those companies, Messrs. Ayres and Barney received orders to pay the claim.

We frequently hear complaints made by those who have met with losses by fire, that Insurance Companies are apt to quibble, and attempt to reduce the sum to be paid by them, to a figure far below what is called for in the policy. Nothing of the sort is ever attempted by the companies above named, provided the claim is just and the proofs correct. The MARKET and the NATIONAL are prompt-paying and reliable companies—patronize them.

---

MICROSCOPIC WONDERS.—Lewenbœck tells us of an insect seen with the microscope, of which twenty-seven million would only equal a mite. Insects of various kinds may be seen in the cavities of a grain of sand. Mould is a forest of beautiful trees, with the branches, leaves and fruit. Butterflies are fully feathered. Hairs are hollow tubes. The surface of our bodies is covered with scales like a fish; a single grain of sand would cover one hundred and fifty of these scales, and yet a scale covers five hundred pores. Through these narrow openings the sweat forces itself like water through a sieve. The mites make five hundred steps a second. Each drop of stagnant water contains a world of animated beings, swimming with as much liberty as whales in the sea. Each leaf has a colony of insects grazing on it like cows in a meadow.

---

A WARNING TO BACHELORS.—Belleford, a distinguished Belgian physician, has recently published a statistical memoir, in which he shows that marriage greatly increases longevity in both sexes. Women who marry at twenty, have a chance of life eleven years greater than that of those who remain single. The probabilities of life for married men exceed those of bachelors by nineteen years. Thus, it appears that from the age of twenty to thirty, the mortality of husbands to bachelors is only as one to twelve, while that of wives to spinsters is one to six for the same period of life. Therefore, ye spinsters and bachelors, take warning and commit matrimony as speedily as possible. No cards.

---

STREET CARS.—Now that our citizens are about to enjoy the luxury of street cars, and as they are likely to be crowded by passengers, particularly while they are new and novel, we will suggest that the most healthful seat to occupy in such a conveyance, is the front of the car, near the driver. The forward motion of the car causes a current of air backward, conveying with it the exhalations from the lungs of the passengers sitting to the front. If these exhalations are flavored with onions, bad whisky, catarrh or consumption, it is not the most pleasant or healthful inhalation a person can take. When entering a street car, sit as far as possible to the front; you will not only secure the most healthful position for yourself, but will also not obstruct the passage of others who desire seats in the same car.

---

GEOLOGICAL.—A bevy of young ladies, belonging to the geological class at the College in this city, recently, under the direction of Prof. Ford, made an excursion to the coal mines at Ralston. Upon their way they met with a "foot print" of some creature, not recognized by Hugh Miller, but the young ladies had *cents* enough to accurately locate the biped in the scale of animated nature, from the impression made by his foot, thus showing their superiority over the great geologist. Later in the day they brought their hammers to bear upon some antiquated geological specimen, spherical in form, and bearing a close resemblance to a human skull. Upon breaking it open they discovered an extinct species of tumor, belonging to a primeval period, a description of which is not laid down in any of our present works on surgery. What further discoveries were made after their entrance to the mines, we were not able to learn, but understand their trip was a very enjoyable one and that they all returned to the College with excellent appetites and increased geological lore.

---

DR. U—D—G—, (we shan't give the rest of his name,) of the Fifth Ward, it is rumored, has been successful in another very delicate case. We believe this is the third successful issue of the kind in the Doctor's experienced practice, and he has reason to feel proud over the uniform success which has crowned his skillful efforts.—*Daily Gazette.*

Glad Charley didn't give our full name, because everybody would then have known who was meant, and that we have a new baby at our house. As it is, we have only received a thousand or so congratulations, and any quantity of invitations to "take a drink" with our friends, and *their* friends,—but invariably at our own expense. Now, this thing has gone on long enough, and we give notice that we refuse to incur any further expense on our son's account, until he is old enough to approve of such a proceeding, and the lavish expenditure of his means. How do we know but that he may repudiate the entire transaction, and cause his innocent parent to shoulder the entire expense of these promiscuous congratulations? We at least call for a-rest-of-proceedings, until he can be consulted upon the subject. In the meantime, our third son is well, and in the enjoyment of his usual good appetite.

---

CLEAN UP.—See that your cellars are not filled with noxious gasses from the decomposition of vegetables or stagnant water. Remove everything of the sort, have the cellar dry, well ventilated, and the walls whitewashed. If this is not seen to, you are constantly rendering your household liable to malignant fevers or dysentery, from the poisonous gasses that are allowed to be generated in your cellars. Also remove all manner of table refuse from your yards. See that your cess-pool is plentifully sprinkled with lime or a solution of carbolic acid. Allow no refuse to remain above ground that is not similarly treated. In this manner contagion may be prevented and sickness rendered less dangerous.

CHINESE THEORY OF DEATH.—The Philadelphia *Medical Times* gives an account of the Chinese theory of sudden death. It says that a telegraph line, about fifteen miles long, having been constructed near Shanghai, the natives supposed that the messages were carried along the wire by devils in the employ of the foreign barbarians. To this they made no objection, until a Chinaman chanced to die suddenly in a house near which stood one of the telegraph poles. It then occurred to another native genius, (an amateur coroner,) that one of the devils had come down from the wire and killed the unfortunate man; whereupon he and his compatriots proceeded to destroy the dangerous apparatus.

WEIGHT OF BRAIN.—Much has been written recently with reference to the weight of the human brain, and many comparisons have been made between the brain of Rulloff, the great criminal, and that of many men eminent in the sciences. The following table will show the weights of brain of the most scientific men with which the world has been familiar:—

| | Age. | Ounces. |
|---|---|---|
| 1. Cuvier, naturalist | 63 | 64.5 |
| 2. Abercrombie, physician | 64 | 63 |
| 3. Spurzheim, physician | 56 | 55.06 |
| 4. Dirichlet, mathematician | 54 | 53.06 |
| 5. De Morny, statesman and courtier | 50 | 53.6 |
| 6. Daniel Webster, statesman | 70 | 53.5 |
| 7. Campbell, Lord Chancellor | 80 | 53.5 |
| 8. Chalmers, celebrated preacher | 67 | 53 |
| 9. Fuchs, pathologist | 52 | 52.9 |
| 10. Gauss, mathematician | 78 | 52.6 |
| 11. Dupuytren, surgeon | 58 | 50.7 |
| 12. Whewell, philosopher | 71 | 49 |
| 13. Hermann, philologist | 51 | 47.9 |
| 14. Tiedemann, physiologist | 80 | 54.02 |
| 15. Hausmann, mineralogist | 77 | 43.3 |
| Averages of 10 distinguished men | 50.70 | 54.07 |
| Averages of 15 distinguished men | 50.80 | 52.9 |

Rulloff's brain weighed 59 ounces. He was a distinguished linguist, and a successful murderer.

COUNTER PRACTICE.—The *Boston Medical and Surgical Journal* calls the attention of apothecaries to an excellent example afforded by one of their own number, in whose shop is conspicuously placed the notice: "We are pharmacists, but not physicians; we dispense medicines, but do not prescribe for diseases."

MICROSCOPIC DETECTION OF CRIMINALS.—One of the very best "detectives" in the service is the microscope. Through its agency human blood can be detected from that of animals, fowls or fishes, frequently enabling the microscopic expert to fasten the guilt of murder upon a criminal, who had accounted for the presence of blood upon his clothing by declaring it to be that of a chicken, or animal, that he had killed the day before. No matter how long the blood has remained dry upon the clothing, or how small the quantity found, it can be easily recognized as belonging to man, animal or fowl. So closely have these observations been made, that many microscopists are able to distinguish the blood of one animal from another, and tell you from what animal it was taken, after submitting it to the microscopic test. Less than two weeks experiment with human and animal blood, under an excellent microscope, has enabled us to distinguish one from the other, without the least difficulty. Such experiments are as valuable to the public as they are fascinating to the student.

